# Distinct Coagulation Phenotypes and Long-Term Neurological Outcomes in Post-Cardiac Arrest Syndrome: A Latent Class Analysis of a 9-Year Single-Center Cohort

**DOI:** 10.3390/jcm15031287

**Published:** 2026-02-05

**Authors:** Sin Young Park, Sang Hoon Oh, Hyo Joon Kim, Han Joon Kim, Jee Yong Lim

**Affiliations:** 1 Department of Emergency Medicine, Seoul St. Mary’s Hospital, The Catholic University of Korea, Seoul 06591, Republic of Korea; suepark2428@gmail.com (S.Y.P.); ohmytweety@catholic.ac.kr (S.H.O.); liebestest@hanmail.net (H.J.K.); hanjoon@catholic.ac.kr (H.J.K.); 2 International Healthcare Center, Seoul St. Mary’s Hospital, The Catholic University of Korea, Seoul 06591, Republic of Korea

**Keywords:** out-of-hospital cardiac arrest, coagulopathy, Antithrombin III

## Abstract

**Background/Objectives:** Post-cardiac arrest syndrome (PCAS) induces systemic ischemia–reperfusion injury accompanied by sepsis-like coagulopathy. This coagulopathy presents heterogeneously, yet distinct coagulation phenotypes and their impact on hypoxic–ischemic brain injury (HIBI) remain poorly defined. We aimed to identify coagulation phenotypes using latent class analysis (LCA) and assess their association with 6-month neurological outcomes. **Methods:** We retrospectively analyzed adult out-of-hospital cardiac arrest (OHCA) patients treated with targeted temperature management (TTM) between 2011 and 2019 from a prospective registry at a tertiary academic center. LCA was performed using coagulation biomarkers measured at admission and 24 h post-return of spontaneous circulation: D-dimer, fibrinogen, antithrombin III (ATIII), platelet count, and PT-INR. The primary outcome was poor neurological outcome (Cerebral Performance Category 3–5) at 6 months. Secondary outcomes included in-hospital mortality and cerebral edema severity assessed by gray-to-white matter ratio (GWR) on brain CT. **Results:** Among 325 patients, LCA identified three phenotypes: Class 1 (Preserved Coagulation, 36.9%), Class 2 (Hypercoagulable State, 41.5%) characterized by elevated D-dimer with preserved fibrinogen and ATIII, and Class 3 (Consumptive Coagulopathy, 21.5%) marked by profound D-dimer elevation with fibrinogen <150 mg/dL and ATIII <60%. Class 3 exhibited the lowest GWR and highest neuron-specific enolase levels. In multivariable analysis adjusting for age, low-flow time, initial rhythm, and lactate, Class 3 independently predicted poor neurological outcome (adjusted OR 4.52; 95% CI 2.15–9.48), whereas Class 2 did not. **Conclusions:** PCAS-related coagulopathy is heterogeneous. A consumptive coagulopathy phenotype identifies a high-risk subgroup associated with severe brain injury and poor long-term neurological outcomes. Early identification of this phenotype may enable targeted prognostication and guide future phenotype-specific interventional strategies.:

## 1. Introduction

Out-of-hospital cardiac arrest (OHCA) is a major public health challenge, with global survival rates remaining disappointingly low despite improvements in the chain of survival [1]. The primary cause of mortality and long-term disability in survivors who achieve return of spontaneous circulation (ROSC) is Post Cardiac Arrest Syndrome (PCAS) [2]. PCAS is a complex pathophysiological state driven by whole body ischemia–reperfusion injury (IRI), which precipitates a systemic inflammatory response syndrome (SIRS) often described as “sepsis-like” [3].

A pivotal but frequently overlooked component of PCAS is the systemic activation of the coagulation system. Following cardiac arrest, the widespread release of tissue factor from damaged endothelium, coupled with the impairment of endogenous anticoagulant pathways (such as the protein C and antithrombin systems), leads to a broad spectrum of coagulopathies [4]. These can range from mild, subclinical activation of clotting factors to fulminant disseminated intravascular coagulation (DIC) [5]. Crucially, this activation of coagulation is not merely a bystander phenomenon; it actively contributes to organ failure. In particular, the formation of microvascular thrombi can occlude the cerebral microcirculation, exacerbating cerebral ischemia even after macro-circulatory flow has been restored—a mechanism known as the “no-reflow” phenomenon [6,7].

Traditionally, clinicians have relied on individual biomarkers such as D-dimer, platelet count, or fibrinogen to assess the severity of coagulopathy. Elevated D-dimer levels, for instance, have been consistently associated with higher mortality and poor neurological outcomes in PCAS patients [8,9]. However, D-dimer is a non-specific marker of fibrin turnover and cannot distinguish between different underlying pathophysiological states [10]. For example, a high D-dimer level can be observed in a hypercoagulable state where natural anticoagulants and substrates are preserved (compensated state), as well as in a consumptive state where essential clotting factors like Antithrombin III (ATIII) and Fibrinogen are critically depleted (decompensated state). In the field of sepsis, identifying such distinct phenotypes has provided new insights into patient heterogeneity and targeted treatments [11]. Yet, this phenotypic approach has rarely been applied to the PCAS population.

Specifically, the role of Antithrombin III (ATIII) in PCAS warrants closer scrutiny. ATIII is a potent endogenous anticoagulant that inhibits thrombin and Factor Xa. Beyond its anticoagulant effects, ATIII possesses anti-inflammatory properties and is crucial for preserving the integrity of the endothelial glycocalyx [12]. The depletion of ATIII may therefore signal a more severe form of endothelial injury and microvascular failure than simple clotting activation [13].

Recent pathophysiological models of PCAS emphasize the role of ‘shock-induced endotheliopathy’ (SHINE) [14]. The systemic ischemia–reperfusion injury degrades the endothelial glycocalyx, a protective layer that regulates vascular permeability and inhibits coagulation [15]. The shedding of this glycocalyx exposes the subendothelial matrix, triggering a massive release of tissue factor and the consumption of endogenous anticoagulants. In the brain, which is highly susceptible to microcirculatory disturbances, this process can lead to diffuse cerebral microthrombosis. Therefore, accurately phenotyping the coagulation status is crucial not only for hematologic management but also for understanding the extent of cerebral microvascular failure.

In this study, we hypothesized that PCAS patients encompass distinct coagulation phenotypes that define their pathophysiological status more accurately than single biomarkers. Using Latent Class Analysis (LCA), a data-driven probabilistic modeling technique, we aimed to: (1) identify and characterize distinct coagulation phenotypes in a high-quality, single-center cohort of OHCA patients treated with Targeted Temperature Management (TTM); and (2) determine whether a specific phenotype, particularly one characterized by the consumption of coagulation factors, is independently associated with severe hypoxic–ischemic brain injury (HIBI) and poor long-term neurological outcomes.

## 2. Materials and Methods

### 2.1. Study Design and Setting

This retrospective observational study utilized data from the prospective TTM registry of Seoul St. Mary’s Hospital, a tertiary academic medical center in Seoul, Republic of Korea. The study period extended from January 2011 to December 2019. This 9-year timeframe was selected because our institution maintained a consistent TTM protocol and a standardized order set for coagulation biomarkers during this period, ensuring high data consistency and completeness. The study was conducted in accordance with the Declaration of Helsinki and was approved by the Institutional Review Board. The requirement for informed consent was waived due to the retrospective nature of the analysis.

### 2.2. Study Population

We screened all adult patients (aged ≥18 years) with non-traumatic OHCA who were admitted to the emergency department, achieved ROSC, and underwent TTM. Exclusion criteria were as follows: (1) cardiac arrest caused by trauma, drowning, or hanging; (2) active hemorrhage or known pre-existing coagulopathy (e.g., hemophilia, thrombocytopenic purpura, or liver cirrhosis classified as Child-Pugh C); (3) patients on therapeutic anticoagulation (e.g., warfarin or DOACs) prior to arrest; (4) death within 24 h of admission, which precluded the assessment of serial biomarker changes; and (5) significant missing data (>50%) for the core coagulation panel at admission. Missing data were handled using available-case analysis with mean imputation for the LPA (Supplementary Table S2). This allowed the inclusion of all participants with partial data in the latent class enumeration.

### 2.3. Post-Cardiac Arrest Care and TTM Protocol

All patients received intensive care according to a standardized protocol based on the current Advanced Life Support (ALS) guidelines [16]. TTM was induced as soon as possible after ROSC using surface cooling devices (Arctic Sun^®^ Energy Transfer Pads, BD, Franklin Lakes, NJ, USA). The target temperature was set at 33 °C, maintained for 24 h, and followed by controlled rewarming at a rate of 0.25 °C per hour until the core temperature reached 37 °C [17]. Sedation and analgesia were maintained with continuous infusions of midazolam (0.05–0.2 mg/kg/h) and remifentanil (0.05–0.2/µg/kg/min) or cisatracurium to prevent shivering. Mean arterial pressure (MAP) was maintained above 65–70 mmHg using fluid resuscitation and vasopressors (norepinephrine) as needed. Mechanical ventilation was adjusted to maintain normocarbia (PaCO_2_ 35–45 mmHg) and normoxia (PaO_2_ 80–120 mmHg). To strictly control the target temperature, an automatic feedback system monitored the core temperature via an esophageal or bladder temperature probe. Shivering was managed using a stepwise protocol utilizing buspirone and magnesium sulfate, followed by sedation and neuromuscular blockade if necessary. All patients underwent continuous amplitude-integrated electroencephalography (aEEG) to detect seizures, which were treated promptly with antiepileptic drugs.

### 2.4. Data Collection and Biomarker Measurement

We extracted demographic and clinical data including age, sex, comorbidities, witnessed status, bystander CPR, initial rhythm, time from arrest to ROSC (low-flow time), and total epinephrine dose. The primary variables of interest were coagulation biomarkers measured at two time points: immediately upon ED admission (0 h) and 24 h after ROSC (24 h). The panel included:

Platelet count (10^3^/uL)

Prothrombin Time (PT) expressed as International Normalized Ratio (INR)

Fibrinogen (mg/dL)—measured using the Clauss method.

D-dimer (µg/mL)—measured using immunoturbidimetric assay.

Antithrombin III (ATIII) activity (%)—measured using chromogenic substrate assay.

### 2.5. Outcome Assessment

The primary outcome was the neurological status at 6 months after cardiac arrest. This was assessed using the Cerebral Performance Category (CPC) scale, ranging from 1 (good performance) to 5 (brain death/death). A poor neurological outcome was defined as a CPC score of 3 (severe disability), 4 (coma), or 5 [18]. Six-month CPC was obtained through structured outpatient follow-up visits for ambulatory survivors and telephone interviews with patients or legal guardians for those unable to attend. For patients who died within 6 months, CPC 5 was recorded. Follow-up was complete in 97.4% of patients. Secondary outcomes included in-hospital mortality and the severity of brain injury, assessed by: (1) peak serum Neuron-Specific Enolase (NSE) levels measured at 24, 48, and 72 h; and (2) the Gray-to-White Matter Ratio (GWR) measured on the initial brain CT scan [19]. NSE was measured using an electrochemiluminescence immunoassay (Roche Diagnostics, Mannheim, Germany); peak NSE was defined as the maximum value among available timepoints. Brain CT was performed at a median of 23 min post-ROSC, and GWR was measured at the basal ganglia level and averaged across standardized regions. Measurements were performed retrospectively by investigators not involved in clinical care.

### 2.6. Statistical Analysis

Latent Class Analysis (LCA): To identify distinct coagulation phenotypes, we performed LCA using tertile-categorized values (Low/Medium/High based on 33rd and 67th percentiles) of D-dimer, Fibrinogen, ATIII, PT (INR), and Platelet count at 0 and 24 h. LCA is a mixture modeling technique that identifies unobserved subgroups (latent classes) within a population based on observed variables [18]. We tested models with 2 to 5 classes. The optimal number of classes was selected based on the lowest Bayesian Information Criterion (BIC) and Akaike Information Criterion (AIC), as well as entropy (indicating classification quality) and clinical interpretability. We used the poLCA package in R for this analysis [20]. Unlike traditional cutoff-based approaches or K-means clustering, LCA is a probabilistic model that assumes the population consists of unobserved (latent) subgroups. This approach allows for the identification of phenotypes based on the pattern of multiple biomarkers simultaneously, rather than relying on arbitrary thresholds for a single marker. This method is particularly advantageous in heterogeneous clinical syndromes like PCAS, where individual biomarkers may fluctuate due to fluid resuscitation or analytical variability.

### 2.7. Comparison and Regression

Baseline characteristics were compared across the identified phenotypes using one-way ANOVA or the Kruskal–Wallis test for continuous variables and the Chi-square test or Fisher’s exact test for categorical variables. To determine the independent prognostic value of the phenotypes, we constructed a multivariable logistic regression model. The model was adjusted for known predictors of outcome: age, witnessed arrest, bystander CPR, initial shockable rhythm, low-flow time, and initial lactate levels. We reported adjusted odds ratios (aOR) with 95% confidence intervals (CI). All statistical analyses were performed using R software (version 4.2.0), and a *p*-value of <0.05 was considered statistically significant.

## 3. Results

### 3.1. Baseline Characteristics of the Core Cohort

**Figure 1:**
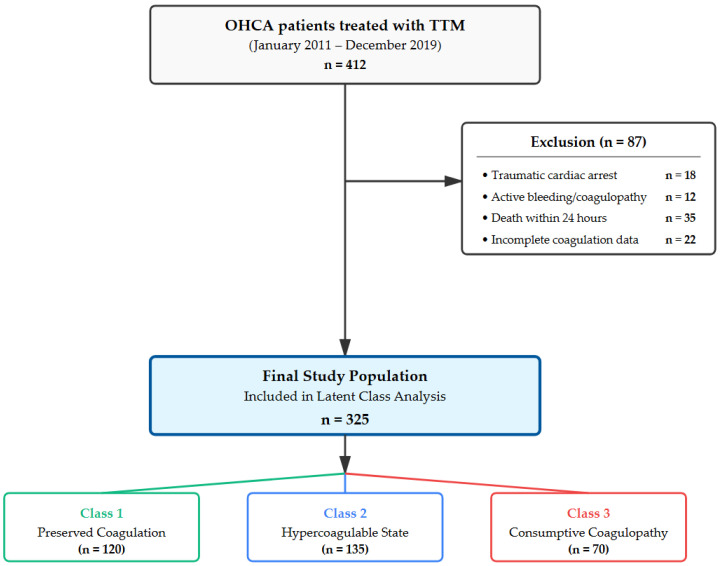
Flow diagram of the included patients. OHCA, out-of-hospital cardiac arrest; TTM, targeted temperature management.

During the 9-year study period, a total of 407 OHCA patients were treated with TTM. After applying exclusion criteria, 325 patients constituted the final core cohort. The mean age was 55.5 ± 16.9 years, and 71.7% (*n* = 233) were male. The etiology of arrest was presumed cardiac in 85% of cases. A shockable rhythm (VF/VT) was the initial rhythm in 123 patients (37.8%). The mean low-flow time was 28.5 ± 15.2 min. At 6 months, 224 patients (69.1%) had a poor neurological outcome (Figure 1 and Table 1).

### 3.2. Identification of Coagulation Phenotypes (Latent Class Analysis)

**Figure 2:**
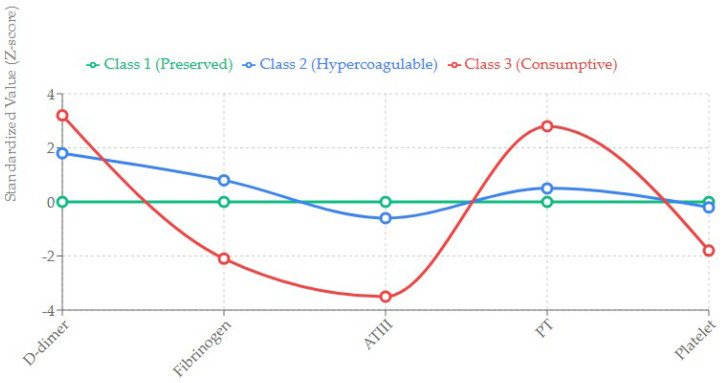
Profiles of Coagulation Phenotypes (Latent Class Analysis).

The LCA model fit statistics (BIC, AIC) indicated that a 3-class model provided the best fit for the data (Supplementary Table S1). The classes were labeled based on their specific biomarker profiles (Figure 2):

Class 1: Preserved Coagulation (*n* = 120, 36.9%)

This group served as the reference. Patients in this class exhibited biomarker levels within or near normal physiological ranges at both 0 and 24 h. D-dimer was mildly elevated (consistent with post-arrest stress), but Fibrinogen and ATIII were well-preserved.

Class 2: Hypercoagulable State (*n* = 135, 41.5%)

This was the largest group. It was characterized by significantly elevated markers of thrombin generation and fibrinolysis (high D-dimer). However, unlike Class 3, these patients maintained normal or even supranormal levels of Fibrinogen and ATIII (activity > 80%). This profile suggests a “compensated” state where the liver can synthesize coagulation factors sufficiently to match consumption.

Class 3: Consumptive Coagulopathy (*n* = 70, 21.5%)

This group exhibited the most severe derangement. D-dimer levels were profoundly elevated (often >35 µg/mL). Crucially, this was accompanied by a sharp decline in substrate availability: Fibrinogen levels dropped below 150 mg/dL, and ATIII activity fell below 60%. PT (INR) was significantly prolonged (>1.5). This pattern is indicative of decompensated coagulopathy or overt DIC.

### 3.3. Temporal Evolution of Biomarkers

Table 2 details the temporal changes in key biomarkers. In Class 3, Antithrombin III levels decreased significantly from admission (52.3 ± 14.5%) to 24 h (41.5 ± 10.2%), reflecting ongoing consumption. In contrast, Class 2 patients maintained stable ATIII levels over 24 h (81.2 ± 11.5%). Similarly, Fibrinogen levels in Class 3 remained critically low, whereas Class 2 showed a trend toward increasing Fibrinogen levels (acute phase reactant response).

### 3.4. Association with Neurological Injury and Outcome

We analyzed markers of brain injury to understand the link between coagulopathy and neurological damage (Table 3).

Brain CT (GWR): Class 3 patients had the lowest mean GWR (1.18 ± 0.05), indicating severe diffuse cerebral edema. In contrast, Class 1 (1.25 ± 0.04) and Class 2 (1.23 ± 0.06) showed relatively preserved gray-white differentiation.

NSE: Peak NSE levels were significantly higher in Class 3 compared to Class 1 and 2 (median 85.4 vs. 32.1 vs. 55.2 ng/mL, *p* < 0.001).

**Figure 3:**
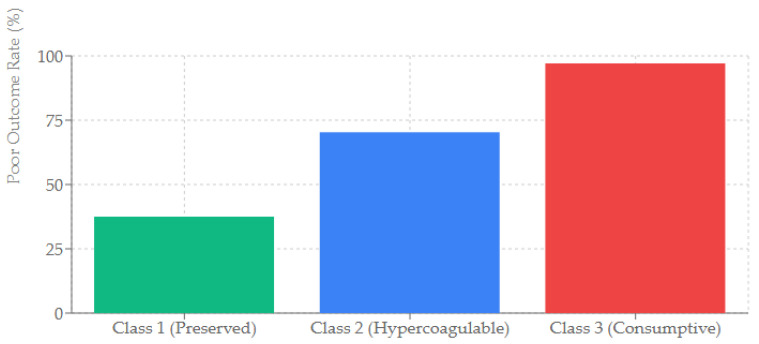
Probability of Poor Neurological Outcome by Phenotype.

The unadjusted rate of poor neurological outcome was 37.5% in Class 1, 70.3% in Class 2, and 97.1% in Class 3 (Figure 3).

### 3.5. Multivariable Analysis

To adjust for potential confounders, a multivariable logistic regression model was performed (Table 4). Factors such as age, low-flow time, initial shockable rhythm, and initial lactate were included. In this adjusted model, Class 2 was no longer a significant predictor of poor outcome (aOR 1.45, 95% CI 0.75–2.85, *p* = 0.215). In stark contrast, Class 3 remained a strong and independent predictor of poor neurological outcome (aOR 4.52, 95% CI 2.15–9.48, *p* < 0.001). This suggests that consumption of coagulation factors carries a specific prognostic weight beyond simple activation or ischemia time.

## 4. Discussion

In this study of 325 OHCA patients treated with TTM, we leveraged Latent Class Analysis to move beyond single-biomarker prognostication. We identified three robust coagulation phenotypes: Preserved, Hypercoagulable, and Consumptive. The most important finding of our study is that the Consumptive Coagulopathy phenotype (Class 3)-defined by the depletion of Antithrombin III and Fibrinogen alongside high D-dimer- is strongly and independently associated with severe hypoxic–ischemic brain injury and poor 6-month neurological outcomes.

Previous studies have largely treated post-arrest coagulopathy as a linear severity scale based on D-dimer or DIC scores [21]. Our results challenge this view. We found that a large proportion of patients (Class 2, 41.5%) had high D-dimer levels but preserved coagulation factors. Importantly, this Hypercoagulable group did not show the same catastrophic neurological prognosis as the Consumptive group (Class 3) in the adjusted analysis. This distinction explains why D-dimer alone has variable specificity in prognostic models [22]. High D-dimer reflects fibrin breakdown, which can occur in both compensated and decompensated states. It is the failure of compensation marked by the exhaustion of ATIII and Fibrinogen that signals a critical tipping point in PCAS pathophysiology. The depletion of Antithrombin III in Class 3 is of particular mechanistic interest. ATIII is not only an inhibitor of thrombin but also a key protector of the endothelial glycocalyx [15]. In the context of whole-body ischemia–reperfusion, the shedding of the endothelial glycocalyx leads to capillary leakage, leukocyte adhesion, and widespread microthrombosis [14]. The low levels of ATIII in Class 3 may reflect consumption due to endothelial injury, although direct markers of glycocalyx damage were not measured in this study.

A critical clinical implication of the Class 3 phenotype is the potential for heparin resistance. Heparin requires Antithrombin III as a cofactor to exert its anticoagulant effect. In patients with Class 3 (Consumptive Coagulopathy), where ATIII activity is profoundly depleted (<60%), the administration of unfractionated heparin—often used during TTM or for VTE prophylaxis—may be ineffective. This could paradoxically leave the cerebral microcirculation unprotected against ongoing thrombosis despite ‘adequate’ dosing. This mechanism highlights why identification of this phenotype is vital; these patients might theoretically benefit from ATIII supplementation rather than increased heparin dosages.

We hypothesize that this systemic coagulopathy may extend to the cerebral microcirculation. The no-reflow phenomenon, where cerebral microcirculation remains obstructed despite patent large vessels, is a major driver of HIBI [23]. Our finding that Class 3 patients had the most severe cerebral edema (lowest GWR) is consistent with—though not direct evidence of—cerebral microvascular dysfunction [24]. These mechanistic inferences remain hypothesis-generating and warrant investigation with direct measurements of microvascular function and endothelial injury markers.

Our findings parallel recent discoveries in sepsis. The Consumptive phenotype we identified is phenotypically similar to the Overt DIC or Coagulopathy phenotype described in sepsis LCA studies [25]. However, in PCAS, the brain is the organ most vulnerable to this microvascular failure. Unlike the liver or kidney, which may recover from temporary ischemia, the brain has little tolerance for no-reflow. Thus, a coagulopathy that might be survivable in sepsis could be fatal in cardiac arrest.

Currently, there is no standard treatment for PCAS-associated coagulopathy. Previous trials of anticoagulants in cardiac arrest have yielded conflicting results, likely because they applied a one-size-fits-all approach [26]. Our study suggests that Class 1 and Class 2 patients, who have preserved physiological reserve, may not benefit from aggressive factor replacement. However, for Class 3 patients, who are in a state of depletion, therapies aimed at restoring the anticoagulant buffer, such as Antithrombin concentrates or Fresh Frozen Plasma (FFP), might be theoretically beneficial [27]. Although the KyberSept trial (Antithrombin in sepsis) failed to show a mortality benefit in the general sepsis population, subgroup analyses suggested benefit in those with DIC without heparin treatment [28]. Given the high mortality of Class 3 PCAS patients, a phenotype-guided trial of ATIII

Several limitations must be acknowledged. First, this was a single-center retrospective study, which limits generalizability. However, the use of a strict TTM protocol and a uniform dataset is a significant strength. Second, we defined phenotypes based on 0 h and 24 h data; a more dynamic model using continuous monitoring could yield finer resolution. Third, we did not directly measure markers of glycocalyx shedding (e.g., Syndecan-1) or perform autopsy studies to confirm cerebral microthrombosis. Fourth, the exclusion of patients who died within 24 h and variable missing rates for coagulation biomarkers may have removed the most critically ill patients. These excluded patients showed more severe coagulopathy at admission (lower ATIII, higher INR), suggesting they would likely have been classified as Consumptive (Supplementary Table S3). This pattern would bias our results toward the null, and our findings likely represent a conservative estimate of the true association. Fifth, our cohort was treated with TTM at 33 °C, which was standard practice during the study period (2011–2019). Since then, the TTM2 trial has demonstrated similar outcomes with targeted normothermia (36 °C), leading to more heterogeneous temperature management strategies. Whether coagulation phenotypes and their prognostic implications differ under normothermia protocols warrants further investigation.

Future studies should investigate whether phenotype-guided resuscitation strategies can improve outcomes. For instance, point-of-care testing (e.g., thromboelastography) could be calibrated to rapidly identify the ‘Consumptive’ phenotype in the emergency department, potentially triggering early specific interventions such as fresh frozen plasma or antithrombin concentrates before irreversible cerebral no-reflow occurs.

## 5. Conclusions

Post-cardiac arrest coagulopathy is clearly heterogeneous rather than a single uniform process. In our analysis, we identified a high-risk consumptive coagulopathy phenotype, defined by concurrent D-dimer elevation with depletion of antithrombin III and fibrinogen. Notably, this phenotype remained independently associated with severe cerebral edema and unfavorable long-term neurological outcomes. These findings suggest that an integrated assessment of coagulation markers may offer clinically meaningful prognostic information and could support the design of future phenotype-guided interventional trials.

## Figures and Tables

**Table 1:** Baseline Characteristics of the Study Population by Coagulation Phenotype.

Variables	Total (*n* = 325)	Class 1 (Preserved) (*n* = 120)	Class 2 (Hypercoagulable) (*n* = 135)	Class 3 (Consumptive) (*n* = 70)	*p*-Value
Age, years (Mean ± SD)	55.5 ± 16.9	52.3 ± 15.2	56.8 ± 17.5	58.9 ± 17.8	0.041
Male Sex, *n* (%)	233 (71.7)	88 (73.3)	95 (70.4)	50 (71.4)	0.876
Witnessed Arrest, *n* (%)	245 (75.4)	95 (79.2)	102 (75.6)	48 (68.6)	0.253
Bystander CPR, *n* (%)	198 (60.9)	78 (65.0)	82 (60.7)	38 (54.3)	0.348
Shockable Rhythm, *n* (%)	123 (37.8)	62 (51.7)	48 (35.6)	13 (18.6)	<0.001
Low-flow time, min (Mean ± SD)	28.5 ± 15.2	22.1 ± 12.5	29.8 ± 14.8	36.9 ± 16.3	<0.001
Initial Lactate, mmol/L (Mean ± SD)	8.2 ± 4.5	6.5 ± 3.8	8.8 ± 4.5	10.2 ± 4.9	<0.001
6-month Poor Outcome, *n* (%)	224 (69.1)	45 (37.5)	95 (70.3)	68 (97.1)	<0.001

**Table 2:** Coagulation Biomarkers at Admission 0 h and 24 h.

Biomarker	Time	Class 1 (Reference)	Class 2 (Hyper)	Class 3 (Consumptive)	*p*-Value
Antithrombin III (%)	0 h	92.5 ± 10.5	85.1 ± 12.2	52.3 ± 14.5	<0.001
	24 h	89.1 ± 9.8	81.2 ± 11.5	41.5 ± 10.2	<0.001
Fibrinogen (mg/dL)	0 h	245 ± 50	310 ± 65	142 ± 45	<0.001
	24 h	260 ± 55	340 ± 70	110 ± 35	<0.001
D-dimer (µg/mL)	0 h	1.5 [0.5–3.2]	18.5 [10.2–25.5]	38.2 [22.1–55.5]	<0.001
PT (INR)	0 h	1.05 ± 0.1	1.15 ± 0.2	1.95 ± 0.6	<0.001

SD, standard deviation. Continuous variables are presented as mean ± SD. *p*-values were calculated using one-way ANOVA or Kruskal–Wallis test as appropriate.

**Table 3:** Comparison of Neurological Injury Markers among Coagulation Phenotypes.

Variable	Class 1 (Preserved) (*n* = 120)	Class 2 (Hypercoagulable) (*n* = 135)	Class 3 (Consumptive) (*n* = 70)	*p*-Value
Brain CT GWR				
Average GWR (Mean ± SD)	1.25 ± 0.04	1.23 ± 0.06	1.18 ± 0.05	<0.001
Basal Ganglia GWR	1.24 ± 0.05	1.22 ± 0.07	1.17 ± 0.06	<0.001
Serum NSE (ng/mL)				
NSE at 24 h (Median [IQR])	32.1 [20.5–45.2]	55.2 [35.1–80.5]	85.4 [50.2–120.5]	<0.001
NSE at 48 h (Median [IQR])	28.5 [18.2–38.5]	65.4 [40.2–95.1]	110.2 [75.5–250.2]	<0.001
NSE at 72 h (Median [IQR])	24.2 [15.5–32.1]	58.1 [30.5–85.2]	95.5 [60.2–180.5]	<0.001

CT, computed tomography; GWR, gray-to-white matter ratio; NSE, neuron-specific enolase; IQR, interquartile range; SD, standard deviation. Continuous variables are presented as mean ± SD for normally distributed data or median [IQR] for skewed data. *p*-values were calculated using one-way ANOVA for GWR and Kruskal–Wallis test for NSE.

**Table 4:** Multivariable Logistic Regression Analysis for Poor Neurological Outcome.

Variable	Crude OR (95% CI)	Adjusted OR (95% CI)	*p*-Value
Age (per 1 year)	1.04 (1.02–1.06)	1.03 (1.01–1.05)	0.012
Low-flow time (per 1 min)	1.08 (1.05–1.11)	1.05 (1.02–1.08)	<0.001
Shockable Rhythm (Yes)	0.25 (0.15–0.42)	0.45 (0.25–0.81)	0.008
Coagulation Phenotype			
Class 1 (Preserved)	Reference	Reference	-
Class 2 (Hypercoagulable)	3.52 (1.85–6.68)	1.45 (0.75–2.85)	0.215
Class 3 (Consumptive)	28.5 (5.5–145.2)	4.52 (2.15–9.48)	<0.001

## Data Availability

The data presented in this study are available on request from the corresponding author. The data are not publicly available due to legal restrictions.

## Supplementary Materials

The following supporting information can be downloaded at: https://www.mdpi.com/article/10.3390/jcm15031287/s1.
