# Supplementary material for: Distinct Coagulation Phenotypes and Long-Term Neurological Outcomes in Post-Cardiac Arrest Syndrome: A Latent Class Analysis of a 9-Year Single-Center Cohort
Source: J Clin Med. 2026 Feb 5;15(3):1287. doi: 10.3390/jcm15031287 (PMC12897979; doi:10.3390/jcm15031287)
Supplement: Supplementary file 1 [file jcm-15-01287-s001.zip › Supplementary_Table_S1.pdf]

Supplementary Table S1. Latent Profile Analysis Model Fit Statistics

Profiles	AIC	BIC	Entropy	Class Sizes (n)
2	5057.24	5279.33	0.916	325, 94
3	4827.76	5162.90	0.861	269, 116, 34
**4**	**3633.25**	**4081.46**	**0.907**	**48, 137, 196, 38**
5	4617.82	5179.09	0.919	77, 122, 25, 46, 149

AIC, Akaike Information Criterion; BIC, Bayesian Information Criterion. Lower AIC/BIC values indicate better model fit. Entropy ranges from 0 to 1, with higher values indicating better classification certainty (>0.80 considered acceptable). The 4-profile solution showed the lowest BIC, while the 3-profile solution was selected for the primary analysis based on parsimony and clinical interpretability.
