# Supplementary material for: Distinct Coagulation Phenotypes and Long-Term Neurological Outcomes in Post-Cardiac Arrest Syndrome: A Latent Class Analysis of a 9-Year Single-Center Cohort
Source: J Clin Med. 2026 Feb 5;15(3):1287. doi: 10.3390/jcm15031287 (PMC12897979; doi:10.3390/jcm15031287)
Supplement: Supplementary file 1 [file jcm-15-01287-s001.zip › Supplementary_Table_S2.pdf]

Supplementary Table S2. Missing Data Pattern by Biomarker and Timepoint (n=428)

Biomarker	Missing at 0h	Missing at 24h
D-dimer	153 (35.7%)	103 (24.1%)
Fibrinogen	75 (17.5%)	85 (19.9%)
Antithrombin III	64 (15.0%)	73 (17.1%)
PT-INR	28 (6.5%)	56 (13.1%)

Values are presented as n (%). Missingness was significantly higher in patients with poor neurological outcomes for D-dimer at 0h (41.4% vs. 19.7%, $p<0.001$) and most 24h biomarkers, suggesting that the most critically ill patients were too hemodynamically unstable for comprehensive testing.
