# Supplementary material for: Distinct Coagulation Phenotypes and Long-Term Neurological Outcomes in Post-Cardiac Arrest Syndrome: A Latent Class Analysis of a 9-Year Single-Center Cohort
Source: J Clin Med. 2026 Feb 5;15(3):1287. doi: 10.3390/jcm15031287 (PMC12897979; doi:10.3390/jcm15031287)
Supplement: Supplementary file 1 [file jcm-15-01287-s001.zip › Supplementary_Table_S3.pdf]

Supplementary Table S3. Characteristics of Patients Who Died Within 24 Hours

Characteristic	Early Death (n=16)	Survived >24h (n=412)	p-value
Age, years	70.5 (59.4-79.5)	55.5 (44.0-68.7)	**0.005**
Male, n (%)	12 (75.0%)	293 (71.1%)	0.956
Witnessed arrest, n (%)	11 (68.8%)	277 (67.9%)	1.000
Bystander CPR, n (%)	9 (56.2%)	248 (60.9%)	0.908
Shockable rhythm, n (%)	8 (50.0%)	228 (55.7%)	0.844
D-dimer at 0h, μg/mL	9.0 (4.9-9.2)	7.6 (4.0-14.6)	0.957
Fibrinogen at 0h, mg/dL	174.0 (140.0-263.0)	220.0 (176.0-275.8)	0.372
ATIII at 0h, %	62.4 (50.0-75.0)	75.0 (64.0-84.3)	**0.043**
INR at 0h	1.5 (1.3-1.9)	1.2 (1.1-1.4)	**<0.001**

Values are presented as median (interquartile range) for continuous variables and n (%) for categorical variables. Early death defined as hospital days ≤1. ATIII, antithrombin III; INR, international normalized ratio. Patients who died early had more severe coagulopathy at admission (lower ATIII, higher INR), suggesting they would likely have been classified as Consumptive phenotype.
